# A Grave Scandal

**Published:** 1903-02-14

**Authors:** 


### The Hospital.

A Journal of

The Medical Sciences and Hospital Administration.

Vol. XXXIII.—No. 855. Edited by Sir Henry Burdett, K.C.B., and Solomon C. Smith, M.D., M.R.C.P. [February 14, 1903.

### A Grave Scandal.

Last week we described the system under which "drunk or dying" cases are at present dealt with at most of the hospitals in London. The same system has been continued for thirty years and more; it has led to grave scandals which have materially damaged the hospitals, and its continuance to the present day is a serious reflection upon every person who is actively connected with the management and direction of the general hospitals of the metropolis. Over and over again the medical and lay Press have demanded a change of system, but in vain. The most serious aspect of the question, too, is that the provision of a few emergency beds at each hospital, where this type of case can be retained under observation, would cost a sum so small as to be inappreciable. Yet at only four of the twelve general hospitals with medical schools has any attempt been made to provide temporary beds for these doubtful cases, and in two of these, St. George's and University College, the accommodation is inadequate. The public has a right to demand why the London, Guy's, St. Thomas's, Middlesex, St. Mary's, King's College, the Westminster, and the Royal Free Hospitals and other general hospitals without medical schools, have not fulfilled their duty to the public in this matter. Within the last month there have been three cases —two are reported on another page of this issue— that affect the credit of St. Mary's, the Middlesex, and St. Thomas's Hospitals, which have been practically censured by a coroner and his jury. As further neglect to provide these emergency beds means probably the sacrifice of life unnecessarily in certain cases, the time has surely come when, in the interests of the hospitals as well as in the interests of humanity and the public, each hospital must choose between at once doing its duty in this matter, or deservedly forfeiting public confidence and public support.

The whole responsibility must be placed upon the managing committees. They are the responsible managers, and to them the public look for the efficient administration of each hospital. The chairman and principal executive officer as representing the committee will one day be brought to book unless the old bad system is immediately altered. It passes our understanding how any committee or official can calmly permit the reputation of their institution to be imperilled by further neglect in this matter.

We have continuously supported the hospitals, and we can claim that few have done more than ourselves to uphold public confidence in the hospitals and to increase their revenues. But every true friend of the voluntary hospital system and of the great hospitals of London must recognise that if the hospitals immediately concerned do not at once provide the simple safeguard of a few emergency beds, to protect the patients from the risks which they are now forced to incur, the remedy must be applied from without. Both the public and the coroners can enforce the immediate provision of emergency beds. The public will be justified in refusing to contribute in money to any general hospital which does not immediately reform its system. One of the most able coroners in a large provincial town effectually stopped these scandals by publicly intimating that on the next "drunk or dying" case coming before him and a jury, if the facts were clearly proved, he would commit the chairman of the committee and the superintendent of the hospital for manslaughter. When it is realised that the resident medical officers, who, though young, are the ablest and best men the schools can provide, would gladly retain every doubtful case if they could induce the authorities to provide the means to enable them to do so, the responsibilities of the committees of management will be recognised. It is wholly wrong for any committee to permit the public to think that any responsibility, in the majority of these cases, attaches to the resident medical staff. The whole responsibility must be placed upon the right shoulders, *i.e.* upon the committee of management, and every hospital committee which allows "drunk or dying" cases to be sent away because it has not provided a few emergency beds deserves the severest condemnation.

We have spoken strongly because the case demands it. Never before in the history of the voluntary hospitals have the public acted so generously or shown such confidence in the management of our voluntary hospitals. This confidence is on the whole abundantly justified by the results, but in certain matters, such as that we are considering, changes are imperatively called for and must be promptly made. The people of London demand that their general hospitals shall be so conducted as to avoid unnecessary suffering and risk to the patients who are brought to them for treatment. They will insist that every facility shall be provided for the medical officers to carry on their work to the best advantage of the patients. There is further justification for a feeling, which is increasing in intensity amongst the public, that the present system of admitting or rejecting in-patients at all the London hospitals requires reconsideration, for at present it leads sometimes to the rejection of certain cases which, though possessing no special interest from a clinical point of view, are distinctly proper and urgent cases for admission when the circumstances of the patient are considered. We should like to see a conference of the committees of all the metropolitan general hospitals called to inquire into this question, with a view to ascertain how far, if at all, public opinion is correct in its present impression, that a number of respectable poor people, sometimes suffering from serious accident, have to find a bed in the Poor Law infirmaries, because the general hospitals do not admit them to in-patient treatment in the present circumstances. Several letters have reached us of late containing complaints on this head, and the sooner the present system of admission is reconsidered by the hospital authorities, the sooner will hospital scandals, with the consequent loss of revenue and popularity be prevented.

